# Overexpression of miR-216b sensitizes NSCLC cells to cisplatin-induced apoptosis by targeting c-Jun

**DOI:** 10.18632/oncotarget.22171

**Published:** 2017-10-27

**Authors:** Gang Huang, Jiongwei Pan, Zaiting Ye, Bingmu Fang, Wei Cheng, Zhuo Cao

**Affiliations:** ^1^ Department of Traditional Chinese Medicine, The Sixth Affiliated Hospital of Wenzhou Medical University, Lishui People’s Hospital, Lishui, 323000, China; ^2^ Department of Respiratory, The Sixth Affiliated Hospital of Wenzhou Medical University, Lishui People’s Hospital, Lishui, 323000, China; ^3^ Department of Radiology, The Sixth Affiliated Hospital of Wenzhou Medical University, Lishui People’s Hospital, Lishui, China, 323000; ^4^ Department of Hematology and Oncology, The Sixth Affiliated Hospital of Wenzhou Medical University, Lishui People’s Hospital, Lishui, 323000, China; ^5^ Affiliated Hospital of Xuzhou Medical University, Jiangsu Province Key Laboratory of Anesthesiology and Center for Pain Research and Treatment, Xuzhou, 221000, China

**Keywords:** NSCLC, miR-216b, c-Jun, cisplatin, apoptosis

## Abstract

Platinum-based chemotherapy is still be the standard treatment for non-small cell lung cancer (NSCLC). Recently, studies demonstrate that some kinds of microRNAs (miRNAs) are associated with chemosensitivity of NSCLC cells to platinum-based treatment. Unfortunately, cancer cells usually change their expression profile of miRNAs to form drug resistance against chemotherapy. In the present study, we focused on miR-216b to investigate whether miR-216b determined sensitivity of NSCLC cells to cisplatin. We observed that expression level of miR-216b was significantly decreased in NSCLC cell lines when they were under the cisplatin treatment. However, restore of miR-216b by transfecting with its mimics was found to increase the cytotoxicity of cisplatin to NSCLC cells. Studies on mechanisms elucidated that miR-216b targeted c-Jun in NSCLC. Overexpression of miR-216b can suppress the cisplatin-induced upregulation of c-Jun. As the downstream, overexpression of Bcl-xl induced by c-Jun/ATF2 heterodimers was inhibited in miR-216b transfected NSCLC cells. Since Bcl-xl is a key anti-apoptotic protein, we found that sensitivity of NSCLC cells to cisplatin-induced apoptosis was significantly increased because of the overexpression of miR-216b.

## INTRODUCTION

According to statistics, non-small cell lung cancer ( NSCLC) represents as the most common cancer worldwide. Because of its high incidence and high metastatic potential, NSCLC is a leading cause of cancer-related deaths. Despite recent advancements in chemotherapy and molecular-targeted therapy, the overall 5-year survival rate is still less than 15% [1, 2]. For patients with advanced NSCLC, platinum-based chemotherapy is still be a standard treatment. However, primary or acquired resistance to chemotherapy severely attenuates the clinical therapeutic effect on patients who are under the platinum-based treatment [3, 4, 5].

Cisplatin is a major platinum-based anti-tumor drug used for NSCLC treatment [6, 7]. Cisplatin in cancer cells damages cellular DNA by forming the cross-link with DNA. Because damage of DNA is a key apoptosis signal, cisplatin exhibits its antineoplastic activity through inducing the apoptotic cell death of cancers [8, 9]. However, cancer cells usually develop mechanisms to protect them from apoptosis induced by cisplatin. For example, expression of c-Jun, an oncogene for promoting cell survival, can be significantly increased when the cancer cells were under the stress of cisplatin [10]. It is urgent to explore strategies to solve the problem of chemoresistance to cisplatin.

Recently, studies demonstrate that some kinds of microRNAs (miRNAs) are associated with chemosensitivity of NSCLC cells to platinum-based treatment. MiRNAs are a class of non-coding, endogenous and small RNAs (20-25 nucleotides in length) in cells. MiRNAs can bind to the 3′-untranslated region (3′ UTR) of target mRNAs, and thus reducing the expression of them through suppressing translation or inducing degradation of mRNAs [11, 12, 13]. Since 60% of the human genes are regulated by miRNAs, it has been found that miRNAs play important roles in cell proliferation, differentiation and apoptosis [14, 15]. Unfortunately, cancer cells including NSCLC usually change their expression profile of miRNAs to survive under the drug-stress [16, 17]. In the present study, we focused on miR-216b to investigate whether miR-216b determined sensitivity of NSCLC cells to cisplatin.

## RESULTS

### Overexpression of miR-216b sensitizes NSCLC cells to cisplatin treatment

**Figure 1:**
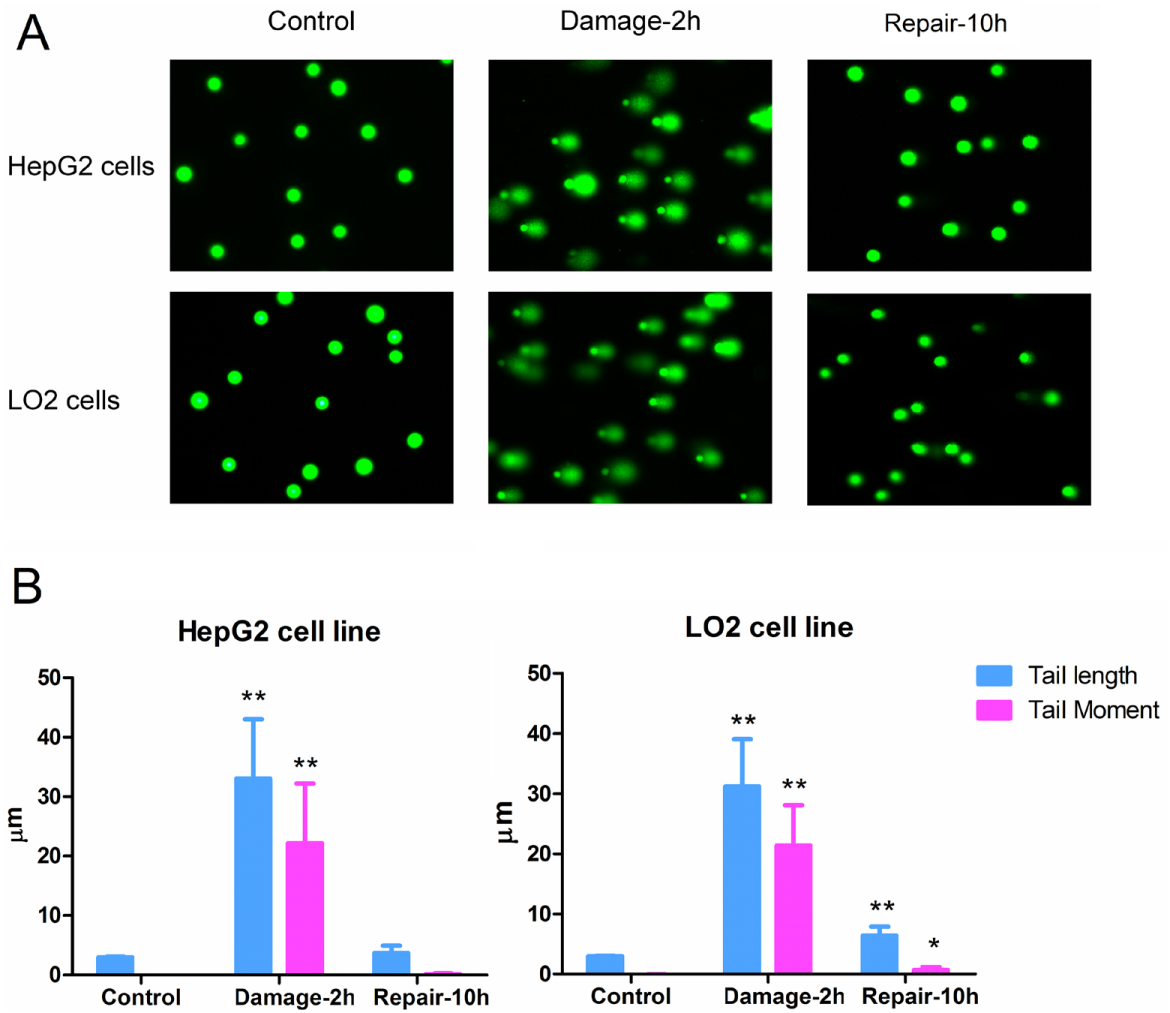
Role of miR-216b in chemosensitivity of NSCLC cells to cisplatin treatment **(A)** QRT-PCR analysis was performed to evaluate the change of miR-216b expression when the A549 and PC9 NSCLC cells were under the cisplatin treatment. ^*^P<0.05. **(B)** Effect of miR-216b mimics (50 pmol/ml) or inhibitors (50 pmol/ml) on changing cellular level of miR-216b in A549 and PC9 NSCLC cells. ^*^P<0.05 *vs.* NCO group. ^#^*P*<0.05 *vs.* cisplatin + NCO group. **(C)** MTT assay was performed to evaluate the effect of miR-216b mimics (50 pmol/ml) or inhibitors (50 pmol/ml) on changing IC50 of cisplatin to A549 cells. ^*^P<0.05 *vs.* NCO group. **(D)** Effect of miR-216b mimics (50 pmol/ml) or inhibitors (50 pmol/ml) on changing IC50 of cisplatin to PC9 cells. ^*^P<0.05 *vs.* NCO group.

Results of qRT-PCR analysis showed that A549 and PC9 NSCLC cells decreased the expression level of miR-216b when they were under the cisplatin treatment (Figure 1A). To explore whether expression profile of miR-216b was associated with chemosensitivity to cisplatin, we transfected the A549 and PC9 cells with miR-216b mimics or inhibitors to change the cellular level of miR-216b by force (Figure 1B). We found that overexpression of miR-216b significantly decreased the 50% inhibiting concentration (IC50) of cisplatin, whereas knockdown of miR-216b enhanced the resistance to cisplatin in A549 cells (Figure 1C) and PC9 cells (Figure 1D). These data indicated that overexpression of miR-216b can elevate the response of NSCLC cells to cisplatin treatment.

### MiR-216b targets c-Jun in NSCLC

**Figure 2:**
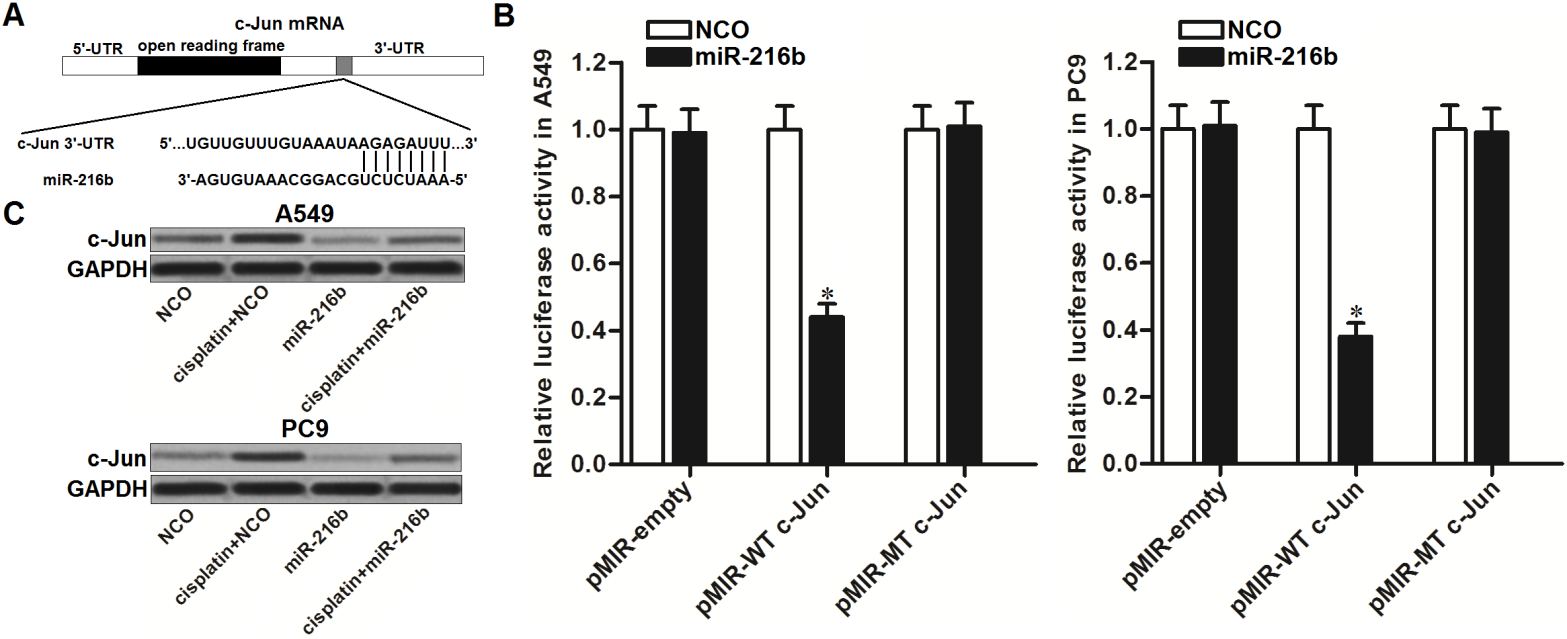
MiR-216b suppresses c-Jun expression in NSCLC **(A)** Putative binding sequence of c-Jun mRNA paired with miR-216b. **(B)** After co-transfection with miR-216b (50 pmol/ml) and pMIR reporters (2 μg/ml) in A549 and PC9 NSCLC cells, relative luciferase activities of pMIR reporters were measured by using Dual-Luciferase Reporter System. ^*^P<0.05 *vs.* NCO group. **(C)** Effect of miR-216b (50 pmol/ml) and cisplatin (2 μM) on changing protein level of c-Jun in A549 and PC9 NSCLC cells.

To explore the mechanism by which miR-216b sensitized NSCLC cells to cisplatin, TargetScan, miRanda, and PicTar public databases were used to predict the potential target of miR-216b in NSCLC. We observed that the oncogene of c-Jun containing putative binding sequence paired with miR-216b at the 3’ UTR of its mRNA (Figure 2A). To confirm that miR-216b targets c-Jun in NSCLC, luciferase reporter assays were performed. The results showed that co-transfection with miR-216b mimics significantly decreased the luciferase activities of pMIR reporters containing wild type (WT) c-Jun 3’ UTR in both A549 and PC9 NSCLC cells. However, miR-216b exhibited no effect on the pMIR reporters containing mutant type (MT) c-Jun 3’ UTR (Figure 2B). We thus demonstrated that miR-216b targets c-Jun in NSCLC. To test the effect of miR-216b on cisplatin-induced upregulation of c-Jun, we detected the protein level of c-Jun in NSCLC cell lines after they were treated with cisplatin and miR-216b. As shown in Figure 2C, single treatment of miR-216b was able to decrease the expression of c-Jun in A549 and PC9 NSCLC cells. Moreover, transfection with miR-216b was found to abolish the upregulation of c-Jun induced by cisplatin. These data indicated that miR-216b suppressed the overexpression of c-Jun in cisplatin-treated NSCLC cells.

### MiR-216b sensitizes NSCLC cells to cisplatin treatment through decreasing the expression of c-Jun

**Figure 3:**
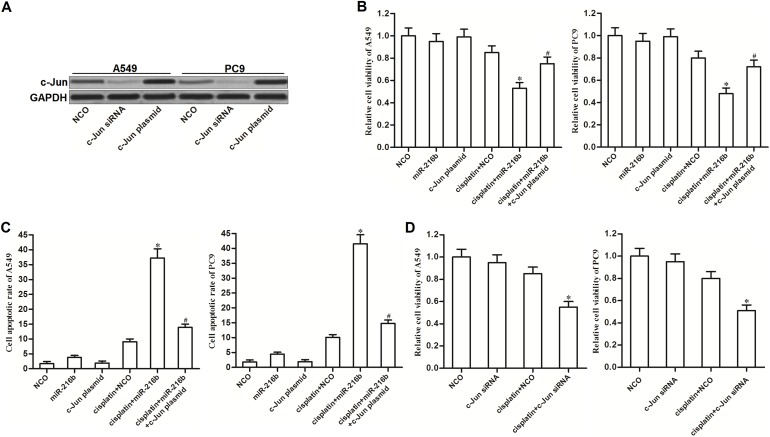
MiR-216b sensitizes NSCLC cells to cisplatin treatment through decreasing the expression of c-Jun **(A)** Western blot analysis was performed to evaluate the effect of c-Jun siRNA (50 pmol/ml) and c-Jun plasmid (2 μg/ml) on changing the cellular protein level of c-Jun in A549 and PC9 NSCLC cells. **(B)** MTT assay was performed to determine the viability of A549 and PC9 cells after they were treated with miR-216b mimics (50 pmol/ml), c-Jun plasmid (2 μg/ml) and cisplatin (2 μM). ^*^P<0.05 *vs.* cisplatin + NCO group. ^#^*P*<0.05 *vs.* cisplatin + miR-216b group. **(C)** After treatment with miR-216b mimics (50 pmol/ml), c-Jun plasmid (2 μg/ml) and cisplatin (2 μM), A549 and PC9 cells were stained with Annexin V followed by detection of cell apoptotic rate on flow cytometry. ^*^P<0.05 *vs.* cisplatin + NCO group. ^#^*P*<0.05 *vs.* cisplatin + miR-216b group. **(D)** Effect of c-Jun siRNA (50 pmol/ml) on cisplatin-induced (2 μM) cytotoxicity to A549 and PC9 NSCLC cells. ^*^P<0.05 *vs.* cisplatin + NCO group.

As c-Jun was targeted by miR-216b, we were supposed to explore whether the miR-216b-sensitized cell death in cisplatin-treated NSCLC cells was dependent on the suppression of c-Jun. We thus overexpressed the c-Jun in A549 and PC9 NSCLC cells by transfection with recombinant expression vector of c-Jun (Figure 3A). Although miR-216b dramatically increased the cytotoxicity of cisplatin to NSCLC cells, enforced expression of c-Jun significantly inhibited the synergistic effect of miR-216b (Figure 3B). Furthermore, we observed that miR-216b significantly enhanced the ability of cisplatin to induce apoptosis of NSCLC cells. However, restore of c-Jun prevented the miR-216b-promoted apoptosis when the NSCLC cells were under the cisplatin treatment (Figure 3C). These results indicated that the miR-216b-sensitized apoptotic cell death in cisplatin-treated NSCLC cells was dependent on the suppression of c-Jun. Next, we knockdown the expression of c-Jun directly in NSCLC cells by transfection with its specific siRNA. We observed that the effect of c-Jun siRNA was similar with miR-216b. C-Jun siRNA treatment also can sensitize NSCLC cells to cisplatin-induced cytotoxicity (Figure 3D). We therefore emphasized the importance of c-Jun suppression in miR-216b-promoted cell death.

### MiR-216b targets c-Jun/Bcl-xl pathway to promote cisplatin-dependent mitochondrial apoptosis in NSCLC

**Figure 4:**
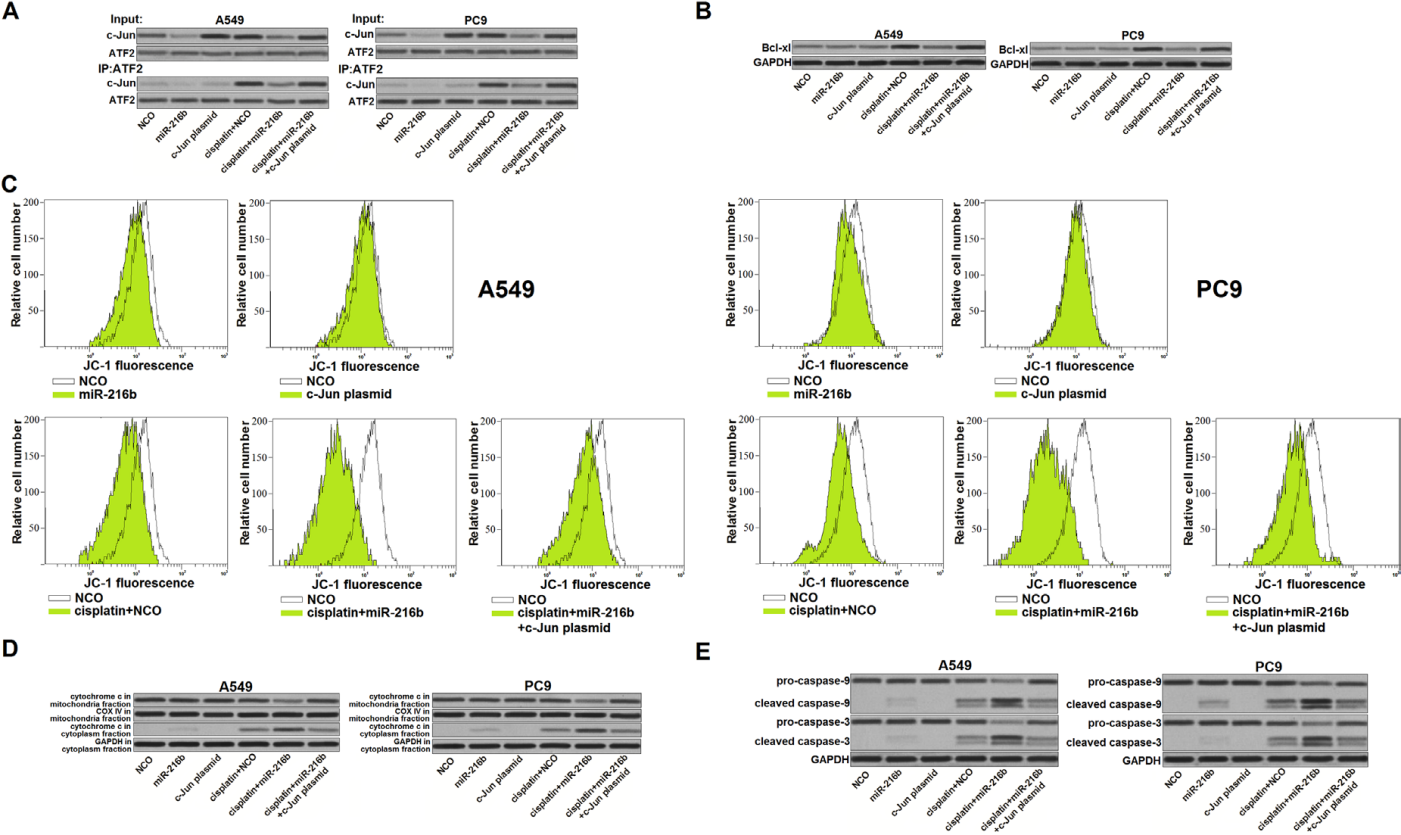
MiR-216b targets c-Jun/Bcl-xl pathway to promote cisplatin-dependent mitochondrial apoptosis in NSCLC **(A)** Interaction with ATF2 and c-Jun in A549 and PC9 NSCLC cells was evaluated by co-immunoprecipitation assay after they were treated with miR-216b mimics (50 pmol/ml), c-Jun plasmid (2 μg/ml) and cisplatin (2 μM). **(B)** Effect of miR-216b mimics (50 pmol/ml), c-Jun plasmid (2 μg/ml) and cisplatin (2 μM) on changing protein level of Bcl-xl in A549 and PC9 NSCLC cells. **(C)** After treatment with miR-216b mimics (50 pmol/ml), c-Jun plasmid (2 μg/ml) and cisplatin (2 μM), A549 and PC9 cells were stained with JC-1 followed by detection of mitochondrial membrane potential on flow cytometry. **(D)** Protein level of cytochrome c in mitochondria fraction and cytoplasm fraction was evaluated by western blot analysis. **(E)** After treatment with miR-216b mimics (50 pmol/ml), c-Jun plasmid (2 μg/ml) and cisplatin (2 μM) in A549 and PC9 cells, cleavage of caspase-9 and -3 was evaluated by western blot analysis.

Since ATF2 is a common co-activator with c-Jun [18], we next explored the effect of miR-216b and cisplatin on the interaction with c-Jun and ATF2. We observed that cisplatin treatment induced significant interaction with c-Jun and ATF2 in NSCLC cells. However, ATF2 failed to link with c-Jun in the cisplatin-treated NSCLC cells when the miR-216b was transfected (Figure 4A). Because of the decrease of c-Jun/ATF2 heterodimers, NSCLC cells failed to upregulate the Bcl-xl expression level even they were under the cisplatin stress [19] (Figure 4B). Due to the suppression of Bcl-xl, a pro-survival protein belongs to Bcl-2 family and functions against mitochondrial apoptosis [20, 21], we found that overexpression of miR-216b obviously enhance the effect of cisplatin on inducing mitochondria collapse in NSCLC cells (Figure 4C). Furthermore, overexpression of miR-216b was found to enhance the cisplatin-dependent opening of mitochondrial permeability transition pore (mPTP), which induced release of cytochrome c from mitochondria into cytoplasm [22] (Figure 4D). In the presence of cytochrome c, the mitochondrial apoptosis markers caspase-9 and -3 [23] were triggered in the miR-216b and cisplatin co-treated NSCLC cells (Figure 4E). Taken together, we demonstrated that overexpression of miR-216b promoted cisplatin-dependent mitochondrial apoptosis in NSCLC by targeting c-Jun/Bcl-xl pathway.

### Overexpression of miR-216b sensitizes NSCLC to cisplatin treatment *in vivo*

**Figure 5:**
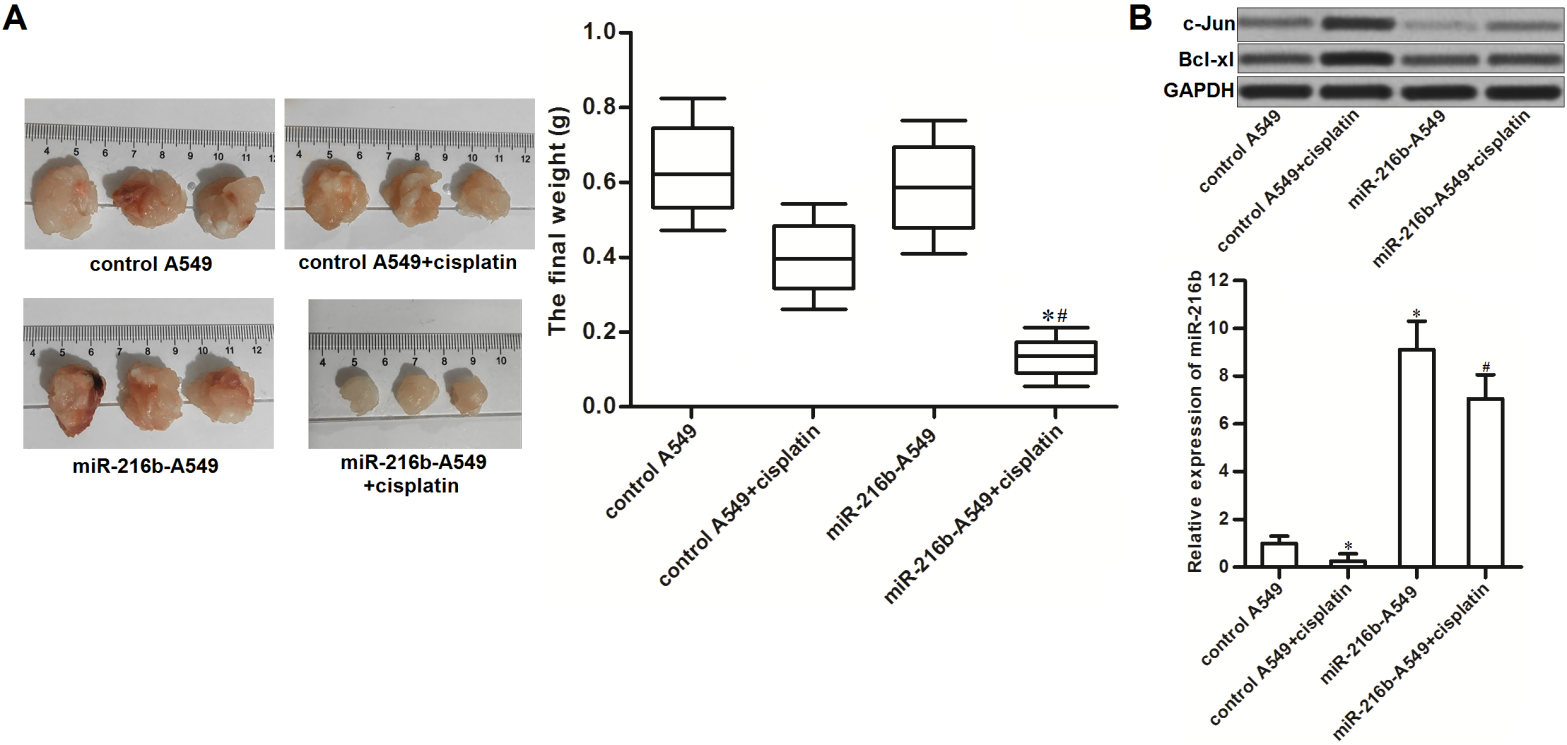
Overexpression of miR-216b sensitizes NSCLC to cisplatin treatment *in vivo* **(A)** Nude mice were inoculated with miR-216b-overexpressed or control A549 cells, followed by administration with cisplatin (2 mg/kg, twice a week). 28 days later, mice were killed and the tumors were resected and weighted. ^*^P<0.05 *vs.* control A549 + cisplatin group. ^#^*P*<0.05 *vs.* miR-216-A549 group. **(B)** Expression levels of c-Jun, Bcl-xl and miR-216b in resected tumors were evaluated by western blot analysis. ^*^P<0.05 *vs.* control A549 group. ^#^*P*<0.05 *vs.* control A549 + cisplatin group.

To explore the potential role of miR-216b in chemosensitivity of NSCLC to cisplatin *in vivo*, we established xenograft tumor model of NSCLC on nude mice by using miR-216b-overexpressed A549 or control A549. We found that the miR-216b-overexpressed NSCLC tumors were obviously smaller than the control ones after they were under the equal dose of cisplatin treatment (Figure 5A). In the resected tumor tissues, the miR-216b-overexpressed tumors expressed obviously lower level of c-Jun and Bcl-xl compared to the control tumors after they were treated with equal dose of cisplatin (Figure 5B). These data suggested that overexpression of miR-216b targeted c-Jun/Bcl-xl pathway to sensitize NSCLC to cisplatin treatment *in vivo*.

### Overexpression of miR-216b sensitizes NSCLC cells to other platinum-based chemotherapeutic drugs

**Figure 6:**
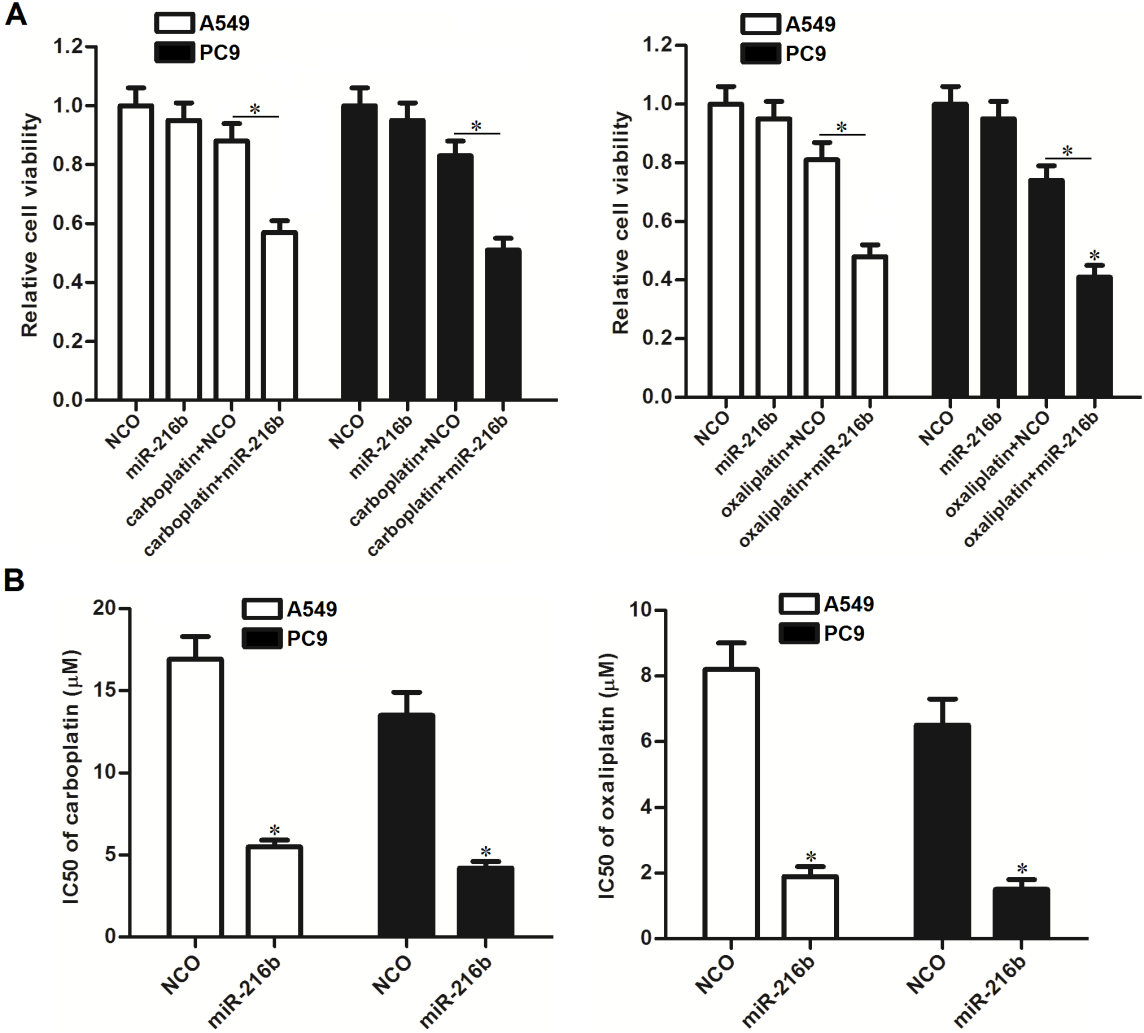
Role of miR-216b in chemosensitivity of NSCLC cells to other platinum-based chemotherapeutic drugs **(A)** A549 and PC9 cells were transfected with miR-216b mimics (50 pmol/ml) followed by treatment with carboplatin (2 μM) or oxaliplatin (2 μM). MTT assay was performed to evaluate the cell viability. ^*^P<0.05. **(B)** Effect of miR-216b mimics (50 pmol/ml) on changing IC50 of carboplatin and oxaliplatin to A549 and PC9 NSCLC cells. ^*^P<0.05 *vs.* NCO group.

To explore whether the sensitization of miR-216b is broad-spectrum on platinum-based chemotherapy, we transfected the A549 and PC9 NSCLC cells with miR-216b mimics, followed by treatment with carboplatin and oxaliplatin. We observed that combination with miR-216b mimics significantly increased the cytotoxicity of both carboplatin and oxaliplatin against A549 and PC9 NSCLC cells (Figure 6A). Overexpression of miR-216b was demonstrated to sensitize NSCLC cells to platinum-based chemotherapeutic drugs, because the IC50 of carboplatin and oxaliplatin was found to be dramatically decreased when the miR-216b was overexpressed by force (Figure 6B). Taken together, these data suggested that the synergistic effect of miR-216b was broad-spectrum on platinum-based chemotherapy in NSCLC.

## DISCUSSION

MiR-216b has been reported to be one of the most frequently downregulated miRNAs in multiple cancers including glioma, melanoma, hepatocellular carcinoma, nasopharyngeal carcinoma and breast cancer [24, 25, 26, 27, 28]. Despite the above studies suggest that miR-216b acts as a potential oncogene in malignant tumors, the role of miR-216b in NSCLC is still unclear. On the other hand, some reports demonstrate that miR-216b increases chemosensitivity in some cancers such as ovarian cancer and hepatocellular carcinoma [29, 30].

As cisplatin is an important chemotherapeutic drug for the treatment of NSCLC, we focused on miR-216b to investigate whether miR-216b determined the sensitivity of NSCLC cells to cisplatin. According to our data, expression level of miR-216b was found to be decreased significantly in NSCLC cells when they were under the cisplatin stress. We therefore speculated that expression profile of miR-216b was associated with cisplatin sensitivity in NSCLC cells. Interestingly, we found that restore of miR-216b decreased the IC50 of cisplatin to NSCLC cells. It indicated that miR-216b can be regarded as a potential sensitizer in cisplatin chemotherapy for NSCLC.

C-Jun is an important member belongs to activation protein-1 (AP-1) transcription family. In cancer cells, c-Jun functions as an important regulator in a wide range of biological processes such as cell proliferation, differentiation, invasion, metastasis and apoptosis [31, 32]. Furthermore, studies report that expression and activation of c-Jun in cancers are highly induced in response to environmental stresses such as damage of DNA [33, 34]. Therefore, cellular c-Jun can be triggered under the platinum-base chemotherapy. Following the overexpression of c-Jun in response to platinum-base chemotherapy, cellular level of complex with c-Jun and activating transcription factor-2 (ATF-2) was highly increased. Subsequently, this complex induces the expression of anti-apoptotic protein Bcl-xl, and thus mediating resistance to apoptosis induced by platinum-base chemotherapy [35, 36, 37]. It has been demonstrated that overexpression of c-jun is responsible for low sensitivity of cancer cells to chemotherapy.

In this study, we found that c-Jun in NSCLC was the target of the miR-216b. Although NSCLC cells upregulated the expression level of c-Jun, which is responsible for chemoresistance, in response to the cisplatin treatment, introduction with miR-216b into NSCLC cells abolished the c-Jun overexpression. Subsequently, NSCLC cells failed to upregulate the cellular level of Bcl-xl, an anti-apoptotic protein downstream of c-Jun pathway, due to the treatment with miR-216b even the cells were under the cisplatin stress. We demonstrated that the miR-216b-dependent suppression of c-Jun/Bcl-xl pathway finally sensitized NSCLC cells to cisplatin-caused mitochondrial apoptosis.

In summary, this study has provided several evidence that overexpression of miR-216b sensitizes NSCLC cells to cisplatin-induced apoptosis by targeting c-Jun. Furthermore, the sensitization of miR-216b is found to be broad-spectrum on platinum-based chemotherapy, because the cytotoxicity of some other platinum-based chemotherapeutic drugs carboplatin and oxaliplatin against NSCLC also can be expanded. However, much more evidence is required to evaluate the potential effect of miR-216b on clinical application of platinum-based chemotherapy in NSCLC.

## MATERIALS AND METHODS

### Cell culture and transfection

Human NSCLC cell lines A549 and PC9 were purchased from the American Type Culture Collection (ATCC, USA). Cells were cultured in RPMI-1640 medium supplemented with 10% FBS and were kept in 5% CO_2_ incubator at 37 °C. For transfection, mature hsa-miR-216b mimics (miR-216b, GenePharma Co. Ltd, Shanghai, China), hsa-miR-216b inhibitors (anti-miR-216b, GenePharma Co. Ltd), negative control oligonucleotides (NCO, GenePharma Co. Ltd), c-Jun small interfere RNA (c-Jun siRNA, Santa Cruz Biotechnology, USA) and recombinant pcDNA3.1 plasmid contained c-Jun open reading frame (c-Jun plasmid, Invitrogen, USA) were introduced into the A549 and PC9 cells by using Lipofectamine 2000 (Invitrogen) according to the manufacturer’s instructions.

### Quantitative real-time PCR

Total RNAs of A549 and PC9 cells were isolated by using Trizol reagent (Invitrogen, USA) according to the manufacturer's instructions. RNAs were converted into cDNA by using One Step PrimeScript miRNA cDNA Synthesis Kit (TaKaRa, China) according to the manufacturer’s protocol. To verify miR-216b expression, Real-time PCR assays were performed by using SYBR Premix Ex Taq (TaKaRa) and the primer (5’-AAATCTCTGCAGGCAAATGTGA-3’) on an ABI PRISM 7900 Sequence Detection System (Applied Biosystems, USA). The mean quantity value of the miR216b expression was normalized to U6 snRNA according to the comparative cycle threshold method (2^-ΔΔCT^) [38].

### Drug sensitivity assay

Transfected A549 and PC9 cells (1×10^4^) were seeded into 96-well plates overnight. Subsequently, culture medium containing platinum-based drugs (1-20 μM) was added to the corresponding cells. 48 h later, 100 μl of MTT working solution (5 mg/ml MTT diluted in the culture medium to 0.5 mg/ml working solution) was added to each well and incubated at 37 °C. 4 h later, The MTT solution was carefully removed followed by addition with and 100 μl DMSO. The absorbance of each well was then read on a spectrophotometer at 570 nm to calculate the relative cell viability. 50% inhibiting concentration (IC50) of cisplatin to A549 and PC9 was determined according to the cell viability curves.

### Luciferase reporter assay

C-Jun 3’ UTR containing predicted wild type miR-216b binding site (WT c-Jun) and corresponding mutant site (MT c-Jun) was cloned into the pMIR-firefly luciferase reporter (Invitrogen). For the luciferase assay, A549 and PC9 cells were co-transfected with pMIR-firefly luciferase reporter, pRL-TK renilla plasmids (Promega, USA) and miR-216b by using lipofectamine 2000. 48 h later, luciferase reporter activities were measured by using a Dual Luciferase Reporter Assay Kit (Promega).

### Western blot analysis

Total proteins were extracted from A549 and PC9 cells by using RIPA buffer (Cell Signaling Technologies, USA) on ice for 30 min. However, For detection of cytochrome c release from mitochondria, the mitochondria fraction and cytoplasm fraction was separated by using Mitochondria/Cytosol Fraction Kit (BioVision, USA). After protein extraction, equal amount of proteins were separated by SDS-PAGE, and then transferred to PVDF membranes. Subsequently, the membranes were blocked with 5% skimmed milk and probed with corresponding primary antibodies. Next, proteins on the membranes were probed with HRP-conjugated secondary antibodies, and then visualized with enhanced chemiluminescent substrate (Thermo Fisher Scientific, Inc, USA).

### Co-immunoprecipitation

Cells were lysed with RIPA buffer on ice for 30 min. Supernatant of cell lysate were incubated with primary antibody of ATF2 (Santa Cruze, USA) at 4 °C overnight before addition with protein G agarose beads. 2 h later, immunoprecipitated pellets were washed three times with RIPA buffer, and then mixed with the SDS loading buffer. After boiling at 95 °C for 5 mins, the immunoprecipitated samples were prepared for the following western blot analysis.

### Detection of mitochondrial membrane potential and cell apoptosis

A549 and PC9 cells were collected and washed with PBS. For evaluation of mitochondrial membrane potential, cells were stained with JC-1 (Molecular Probes, USA) and then analyzed on flow cytometry (Becton Dickinson, USA) to detect the red fluorescence which is indicative of high mitochondrial membrane potential [39]. For detection of cell apoptosis, cells were stained with Annexin V-FITC (Sigma Aldrich, USA) and then analyzed on flow cytometry to measure the apoptotic cells which is the Annexin V-positive population.

### Mouse xenograft models

Recombinant lentivirus contained miR-216b precusor sequence were purchased from the Genechem Co., Ltd. A549 cells were transfected with the above recombinant lentivirus or control lentivirus to prepare the miR-216b-overexpressed A549 (miR-216b-A549) or control A549. BALB/c nude mice were purchased from the Shanghai Super-B&K Laboratory Animal Corp., Ltd. (Shanghai, China) and maintained in a laminar airflow cabinet in a pathogen-free environment. For inoculation, 5×10^6^ prepared A549 cells were subcutaneously injected. Mice were then treated with cisplatin i.p. twice a week (2 mg/kg) before sacrifice by euthanasia on day 28 post-injection. Primary tumors were harvested from mice, and digested by using collagenase type III for the following western blot analysis. Our experimental protocols and animal care were approved by the Animal Care Committee of The Sixth Affiliated Hospital of Wenzhou Medical University/Lishui People’s Hospital.

### Statistical analysis

Data were obtained from at least 3 independent experiments and represented as mean ± SD. Differences between groups were analyzed by ANOVA methods using SPSS 15.0 software. Differences were considered significant when the *p* value was less than 0.05.
